# Multimodal treatment according to the NPC‐GPOH trials in adult patients with nasopharyngeal cancer—Analysis based on a single‐center experience

**DOI:** 10.1002/cnr2.2111

**Published:** 2024-08-27

**Authors:** Martin Leu, Hanibal Bohnenberger, Manuel Guhlich, Markus Anton Schirmer, Yiannis Pilavakis, Hendrik Andreas Wolff, Stefan Rieken, Leif Hendrik Dröge

**Affiliations:** ^1^ Department of Radiotherapy and Radiation Oncology University Medical Center Göttingen Göttingen Germany; ^2^ Institute of Pathology University Medical Center Göttingen Göttingen Germany; ^3^ Department of Otorhinolaryngology University Medical Center Göttingen Göttingen Germany; ^4^ University Medical Center Göttingen Göttingen Germany; ^5^ Department of Radiology Nuclear Medicine and Radiotherapy, Radiology Munich Munich Germany; ^6^ Department of Radiotherapy and Radiation Oncology University Medical Center Regensburg Regensburg Germany

**Keywords:** antiviral treatment, interferon‐β, nasopharyngeal cancer, NPC‐GPOH trials, radiochemotherapy, WHO histological type

## Abstract

**Background and Aim:**

The German NPC‐GPOH trials introduced treatment including neoadjuvant chemotherapy, radiochemotherapy (RCT) and antiviral treatment in patients aged 25 years or younger with nasopharyngeal cancer (NPC). We conducted a retrospective study on outcomes of patients at the age of ≥26 years treated accordingly at our institution.

**Methods:**

Consecutive patients who received primary RCT for NPC were included. The Kaplan–Meier method was used to calculate survival probabilities, and the Cox regression analysis was used to test for an influence of the variables on outcomes. Acute and late toxicity were evaluated via CTCAE criteria and LENT/SOMA criteria, respectively.

**Results:**

In total, 30 patients were included. Diagnosis was made from 09/1994 to 11/2016. The median 5 year overall survival (OS), disease‐free survival (DFS), cancer‐specific survival (CSS) and locoregional recurrence‐free survival (LRC) were 75%, 56%, 83%, and 85%, respectively. We found a negative impact on outcomes (*p* < .05) in case of older age (OS), history of smoking (OS), and T4 stage/ UICC stage IV (DFS). WHO histologic type significantly influenced outcomes, with best outcomes for type III and worst outcomes for type I. The rates of acute and late toxicities were acceptable.

**Conclusion:**

We found excellent outcomes and good feasibility of the NPC‐GPOH trials regimen in adult patients. Additionally, we identified patients with outcomes which need to be improved (smokers, histologic type I tumors) and with particularly excellent outcomes (histologic type III tumors). This stimulates further studies on treatment intensification or de‐escalation aiming at reduced side effects with optimal tumor control in NPC.

## 1 INTRODUCTION

Nasopharyngeal cancer (NPC) rarely occurs in Western Europe.^1^ There are distinct differences between NPC in childhood or adolescence and NPC in adulthood: adults present with a less advanced stage at diagnosis and, paradoxically, have a worse prognosis.^2^, ^3^ The World Health Organization (WHO) classification comprises keratinizing squamous cell carcinoma (type I) and nonkeratinizing carcinoma, which includes differentiated (type II) and undifferentiated carcinoma (type III).^4^ A high percentage of nonkeratinizing carcinomas are Epstein–Barr virus (EBV)‐associated. Both nonkeratinizing type and the presence of EBV are positive prognostic factors.^5^, ^6^

Primary radiochemotherapy (RCT) represents the standard treatment for NPC.^7^, ^8^, ^9^, ^10^, ^11^ Recognizing the distinct features of the EBV‐associated carcinomas, the NPC‐91‐GPOH trial and, subsequently, the NPC‐2003‐GPOH/DCOG trial, included adjuvant antiviral treatment with interferon‐β (IFN‐β) after RCT in children and adolescents. The authors reported excellent outcomes and an acceptable toxicity profile.^7^, ^12^, ^13^

Based on the results, we started to treat all patients in accordance with these protocols. With respect to aforementioned known differences between NPC in childhood or adolescence and adulthood, it is worth investigating whether this regimen can achieve similar results in adults. Therefore, we analyzed outcomes of NPC patients at the age of 26 years or older who were consecutively treated at our institution. Results of a subgroup of this patient cohort were previously published by Wolff et al. with special focus on type III tumors and IFN‐β treatment.^14^ In this study, we report outcomes with an updated and extended patient cohort and analyze the influence of patient‐ and treatment‐related variables.

## 2 PATIENTS AND METHODS

### 2.1 Patients

We included patients who were treated with primary RCT for NPC of WHO type I–III (description of subtypes in accordance with the 1991 WHO classification^4^, ^15^) at the age of 26 years or older. Patients were excluded in case of distant metastases. Patients were staged with upper endoscopy, tumor biopsy (including testing for the presence of EBV according to local standards), computed tomography scan of the head and neck region, and assessment of lung and liver (either using a chest radiograph and ultrasound examination or computed tomography). This investigation was approved by the local ethics committee of the University of Göttingen Medical Center (application number 9/6/19). It was conducted in accordance with the Declaration of Helsinki principles.

### 2.2 RCT and immunotherapy

Radiation therapy was delivered to the primary tumor and to the bilateral neck. Patients were immobilized using a customized thermoplastic mask. Treatment techniques were: 2D planning from 1994 to 1999, 3D planning from 1999 to 2008, and intensity modulated radiotherapy from 2008 (details of planning techniques were described before by Wolff et al.^16^). Based on the respective trials, concomitant chemotherapy was applied starting in the 1990s, while induction chemotherapy was used first in 2003, based on the results of the NPC‐91‐GPOH trial.^7^, ^12^, ^17^ The NPC‐91‐GPOH trial used an induction chemotherapy with 3 cycles of methotrexate, cisplatin, and 5‐fluorouracil, followed by radiotherapy up to a total dose of 59.4 Gy.^12^ The subsequent NPC‐2003‐GPOH/DCOG 2012 trial (in cases of UICC stages III‐IV) included an induction chemotherapy with 3 cycles of cisplatin, 5‐fluorouracil, and folinic acid, followed by RCT (with concomitant cisplatin [20 mg/m^2^/d on 3 consecutive days, during the first and last week of irradiation]) up to a total dose of 59.4 Gy (in cases of complete remission after induction chemotherapy: 54 Gy).^7^ In both studies, adjuvant IFN‐β (10^5^ IU/kg, 3 days per week) was applied for a period of 6 months.^7^, ^12^

According to local practice, in case of medical contraindications, chemotherapy was omitted. After RCT, adjuvant immunotherapy was given in patients with WHO type I and II tumors only in case of positivity for EBV and in patients with WHO type III tumors. The decision for IFN‐β application was made on an individual basis by an interdisciplinary team of the University of Göttingen Medical Center. Patients received thorough information and were informed about the individual character of the treatment before giving informed consent.

### 2.3 Follow‐up and toxicity

During RCT, patients were examined weekly. Acute toxicity was assessed using CTCAE criteria.^18^ The medical records were evaluated. Toxicities were clinically judged according to the current CTCAE classification v5.0.^19^ After RCT, follow‐up was conducted in cooperation with the local department of otorhinolaryngology. Regular follow‐up examinations included upper airway endoscopy and computed tomography scans of the head and neck. Intervals were left at the discretion of the treating radiation oncologist or otorhinolaryngologist. Late toxicity was evaluated using the LENT/SOMA criteria.^20^

### 2.4 Statistics

The survival times were calculated starting from the day of histopathological diagnosis. For the respective definitions see Birgisson et al. (here defined for case of colorectal cancer).^21^ Locoregional control (LRC) was defined as the time to recurrence of primary tumor or recurrence in cervical lymph nodes. Disease‐free survival (DFS) was defined as the time to locoregional recurrence, distant metastases, second primary cancer, or death from any cause. Cancer‐specific survival (CSS) was defined as the time to death from NPC. CSS events only included deaths specifically caused by NPC progression. Deaths due to other causes (e.g., unknown cause, deaths from toxicities) were censored at that time. The Kaplan–Meier method was used to calculate survival rates. The Cox regression analysis was used to test for an influence of the variables on outcomes, using a cut‐off value of *p* < .05. Due to the small sample size, we decided for bivariate categorization of cT stage and AJCC stage. Jen et al. proposed a grouping system for NPC which integrated cT stage and AJCC stage by means of recursive partitioning analysis.^22^ Here, cT4 stage resulted in assignment to the most unfavorable group.^22^ Additionally, AJCC stage IV was associated with considerably lower survival (5 year overall survival (OS), 73.7%) when compared to stages I–III (5‐year OS, 89.2%–94.0%).^22^ Thus, as a pragmatic approach for the presented study, the categorization (cT1‐3 vs. cT4 and AJCC stages IV vs. other stages) was used for survival analysis. Data were analyzed using the software STATISTICA (v13.3, TIBCO Software Inc.) and SPSS Statistics (v26, IBM). The survival curves were drawn using the software R (version 4.3.3) with the plugin KMWin (version 1.53).

## 3 RESULTS

### 3.1 Patients and treatment

We screened 42 patients who were treated for NPC in our clinic. In total, 30 patients were included for further analysis (see Figure S1). Diagnosis was made between 09/1994 and 11/2016. The median follow‐up was 52.0 months (range, 1.9–103.8). For details of patient and treatment characteristics, see Tables 1 and S1 (EBV status, IFN‐β application and histologic type). The induction chemotherapy (*n* = 14) consisted of three cycles of cisplatin (100 mg/m^2^/d on day 1), 5‐fluorouracil (1000 mg/m^2^/d on days 1–5), and folinic acid. The concomitant chemotherapy consisted of cisplatin 6 mg/m^2^/d (daily during irradiation, *n* = 4), cisplatin 20 mg/m^2^/d (given on 3 consecutive days, during the first and last week of irradiation, *n* = 12), cisplatin 20 mg/m^2^/d (given on 5 consecutive days, during the first and fifth week of irradiation, *n* = 2), cisplatin 100 mg/m^2^/d (days 1, 22 and 43 of irradiation, n = 1), carboplatin 100 mg/m^2^/d (given on 3 consecutive days, during the first and last week of irradiation, *n* = 5), and 5‐fluorouracil 600 mg/m^2^/d combined with cisplatin 20 mg/m^2^/d (days 1–5 and days 29–33 of irradiation, *n* = 3).

**TABLE 1:** Baseline patient and disease characteristics.

Patient and disease characteristics	All eligible patients (*n* = 30)
Age (years), median (min–max)	55 (26–81)
Female	9 (30.0)
Male	21 (70.0)
Smoking w/o regular alcohol	8 (26.7)
Smoking and alcohol abuse	3 (10.0)
Neither smoking nor regular alcohol	18 (60.0)
Smoking/alcohol, undetermined	1 (3.3)
cT1	3 (10.0)
cT2	6 (20.0)
cT3	7 (23.3)
cT4	14 (46.7)
cN0	8 (26.7)
cN1	3 (10.0)
cN2	17 (56.6)
cN3	2 (6.7)
Stage II^a^	3 (10.0)
Stage III^a^	13 (43.3)
Stage IV^a^	14 (46.7)
EBV status, negative	9 (30.0)
EBV status, positive	21 (70.0)
WHO type I^b^	5 (16.7)
WHO type II^b^	13 (43.3)
WHO type III^b^	12 (40.0)
Concomitant chemotherapy, no	3 (10.0)
Concomitant chemotherapy, yes	27 (90.0)
Concomitant chemotherapy, incomplete (<100%)	2 (7.4)
Radiotherapy, incomplete (<100%)	2 (6.7)
Radiotherapy technique, 2D/3D	10 (33.3)
Radiotherapy technique, IMRT/VMAT	20 (66.7)
Planned radiotherapy dose (median [min, max])	66.3Gy (54Gy‐70.4Gy)
Applied radiotherapy dose (median [min, max])	66.0Gy (48Gy‐70.4Gy)
Radiotherapy dose per fraction, 1.8Gy	11 (36.7)
Radiotherapy dose per fraction, 2.0Gy	15 (50.0)
Radiotherapy dose per fraction, 2.2Gy	4 (13.3)
Induction chemotherapy, no	16 (53.3)
Induction chemotherapy, yes	14 (46.7)
Induction chemotherapy, incomplete (<100%)	5 (35.7)
Adjuvant interferon, no	21 (70.0)
Adjuvant interferon, yes	9 (30.0)
Adjuvant interferon, incomplete (<100%)	1 (11.1)

*Note*: The number of patients and the percentage (in brackets) or the median and range (in brackets) are given for each parameter, respectively.

^a^ AJCC, 7th edition.

^b^ 1991 WHO classification.

### 3.2 Outcome

**FIGURE 1:**
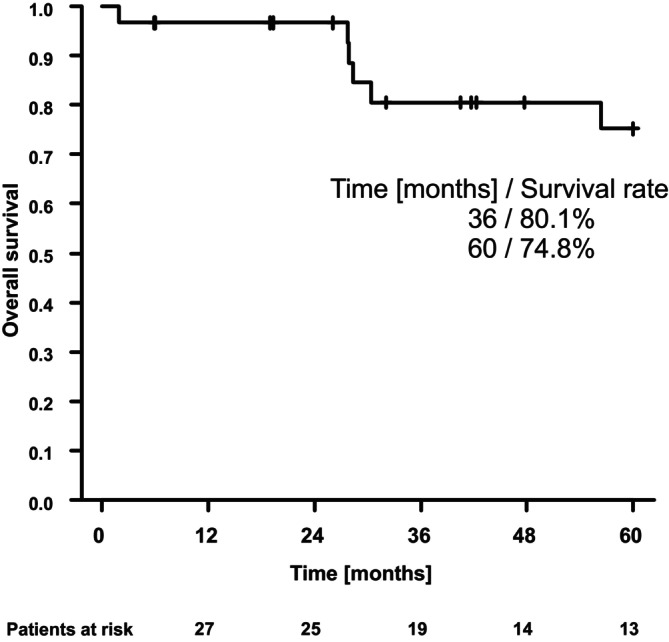
Overall survival for the whole patient cohort.

The median 5 year OS, DFS, CSS, and LRC rates were 75%, 56%, 83%, and 85%, respectively (See Figures 1 and [Link], [Link]). There were three events of locoregional recurrences: one patient experienced local recurrence and two patients experienced cervical lymph node recurrences. Distant metastases occurred in six patients. A total of 11 patients died during follow‐up. Among them, five patients died due to NPC. The remaining causes of death were unknown. In three patients, second primary tumors occurred during follow‐up (basalioma, breast cancer, and leukemia).

We found a negative impact on outcomes (*p* < .05) in case of older age (OS), history of smoking (OS), and T4 stage/ UICC stage IV (DFS). Furthermore, WHO histologic type significantly influenced outcomes, with best outcomes for type III and worst outcomes for type I (DFS, OS; Table 2).

**TABLE 2:** Influence of baseline patient, disease, and treatment characteristics on outcomes.

	DFS		OS	
Variable	Hazard ratio (95% CI)	*p*‐value	Hazard ratio (95% CI)	*p*‐value
Age (per year)	1.03 (0.99–1.07)	.118	1.06 (1.00–1.12)	0.045
History of smoking, yes (18) vs. no (11)	2.76 (0.92–8.27)	.070	7.08 (1.35–37.00)	0.020
cT4 (14) vs. cT1‐T3 (16)	3.51 (1.17–10.60)	.026	2.54 (0.63–10.20)	0.168
Stage IV (14) vs. II + III (16)^a^	3.51 (1.17–10.60)	.026	2.54 (0.63–10.20)	0.188
Induction chemotherapy, complete (9) vs. none (16)	0.70 (0.18–2.67)	.602	0.68 (0.13–3.58)	0.651
Adjuvant IFN‐β, yes (9) vs. no (21)	0.36 (0.08–1.60)	.178	0.33 (0.04–6.70)	0.297
WHO type II + III (25) vs.I (5)^b^	0.30 (0.10–0.87)	.027	1.32 (0.16–10.84)	0.796
WHO type III (12) vs.I + II (18)^b^	0.25 (0.07–0.91)	.035	0.15 (0.02–1.25)	0.079

*Note*: The results of the univariable Cox regression analysis are given (hazard ratios and 95% confidence intervals). We used a cut‐off value of *p* < .05.

Abbreviations: CSS, cancer‐specific survival; DFS, disease‐free survival; IFN‐β, interferon‐β; LRC, locoregional control; OS, overall survival.

^a^ AJCC, 7th ed.

^b^ 1991 WHO classification.

### 3.3 Toxicity

In the whole patient cohort, 16 patients experienced high‐grade (≥grade 3) acute organ toxicity. Eight patients experienced relevant late toxicity (≥grade 2). See Tables 3 and 4 for further details. Three patients had a feeding tube prior to RCT. Additionally, during the course of treatment, 11 patients received a feeding tube. The reasons for incomplete application of the different treatment modalities (percentages of patients: see Table 1) were: fever and adverse gastrointestinal effects (IFN‐β application incomplete, *n* = 1), systemic progression under RCT (radiotherapy and concomitant chemotherapy incomplete, *n* = 1), by patient request due to grade 1 mucositis (radiotherapy incomplete, *n* = 1), and acute kidney injury (chemotherapy incomplete, *n* = 1).

**TABLE 3:** Depiction of the number of patients who experienced acute toxicities.

	*N* (%)
Mucositis	
I°	8 (26.7)
II°	16 (53.3)
III°	5 (16.7)
Dermatitis	
I°	14 (46.7)
II°	13 (43.3)
Dysphagia	
I°	3 (10.0)
II°	5 (16.7)
III°	16 (53.3)

**TABLE 4:** Depiction of the number of patients who experienced late toxicities.

	*N* (%)
Xerostomia	
I°	17 (56.7)
II°	5 (16.7)
III°	2 (6.7)
Hoarseness	
I°	2 (6.7)
II°	1 (3.3)
Dysgeusia	
I°	8 (26.7)
II°	3 (10)
Lymphedema	
II°	2 (6.7)
Fibrosis	
I°	1 (3.3)

## 4 DISCUSSION

Recent trials established a sequence of induction chemotherapy, RCT and IFN‐β application as a highly effective treatment for NPC in children and adolescents.^3^, ^7^, ^12^, ^13^ Accordingly, we introduced this treatment regimen into our clinic for adults. The incidence of NPC has a bimodal age distribution.^3^ Since the aforementioned studies excluded patients in the second peak of NPC incidence (fifth decade^3^), there is less known about treatment results in this patient group. Thus, in this study we present an updated analysis of this special patient cohort treated in our clinic. Especially since most of the patients were diagnosed in advanced tumor stages, our 5 year OS and CSS rates of 75% and 83%, respectively, represent excellent outcomes. These are comparable with previous reports from our institution and from the German study group.^7^, ^12^, ^14^

To further improve outcomes, recently, the specific components and sequences of multimodal treatment have been intensively studied and discussed.^11^, ^23^ The concomitant chemotherapy is very well established.^11^ Accordingly, it was given to the vast majority of patients (90%) in our study. During follow‐up, distant metastases occurred in a relevant percentage of patients (20%), which can be compared with the rates reported in current literature.^11^ Additionally, we found a negative influence of advanced tumor stages on outcomes. Both findings emphasize the need for a further treatment optimization or an escalation of systemic treatment. The induction chemotherapy represents a possible approach. Since recent studies indicate an improvement in oncologic outcomes and, simultaneously, possibly higher toxicity rates and reduced compliance, the role of the induction chemotherapy is still subject of discussion in current literature.^11^, ^24^, ^25^, ^26^, ^27^ In our study, approximately half of the patients received this additional treatment. However, we could not demonstrate a clear benefit of an induction chemotherapy, possibly due to the small sample size.

As a further approach to improve treatment results, the German NPC‐GPOH trials started to treat adolescent patients with IFN‐β in 1992. As Wolff et al. reported in 2010 for parts of the presented cohort treated in our clinic,^14^ the integration of IFN‐β into multimodal treatment strategy yields excellent results and does not relevantly affect treatment tolerability. Now, we additionally tested for an influence of IFN‐β application on outcomes, and, however, firm conclusions cannot be drawn due to the small sample size. To our knowledge, probably due to the rarity of the disease, further comparable data about antiviral treatment in NPC are very rare.^7^, ^12^, ^14^, ^28^ In general, treatment approaches which address different components of the immune system gained relevant interest and should be studied in the near future.^3^, ^29^ Furthermore, it is known that histologic type is a relevant prognostic factor in NPC.^6^, ^30^ The NPC‐GPOH trials included patients with WHO type II and III tumors and reported outcomes for the whole patient cohort, irrespective of histological type.^7^, ^12^ Other previous studies reported a poor prognosis for WHO type I tumors.^6^, ^30^ This could be confirmed in our study. So far, in case of comparison of type II and type III tumors, a consistent prognostic difference could not be demonstrated.^6^, ^31^ In our study, we found that patients with type III tumors had a significantly better prognosis than patients with type I–II tumors. It is known that type III tumors are more closely associated with an EBV infection.^2^, ^12^ Additionally, there is evidence that this implies both an enhanced sensitivity to RCT and to antiviral treatment.^32^, ^33^ Thus, the biological tumor characteristics might plausibly explain the favorable prognosis of type III tumors. Interestingly, considering virus‐associated carcinomas, a similarly favorable prognosis is known for human papillomavirus‐associated oropharyngeal cancers.^34^ This highlights the special characteristics of these tumors in the head and neck region and points out to the need to develop particular strategies for therapeutic management. In case of NPC, these strategies could include the use of pre‐treatment EBV viral load, which was demonstrated to be a useful prognosticator,^35^ and the use of EBV viral load after primary RCT for follow‐up or to guide adjuvant treatment.^36^ In our study, firm conclusions cannot be drawn concerning the effect of IFN‐β on outcomes, putatively due to the retrospective design and small sample size. Previous studies demonstrated that patients with high EBV viral load are at increased risk of recurrence and death.^36^ Thus, the need for adjuvant treatment (e.g., with IFN‐β) might be high in this patient group. However, further studies are required to test this hypothesis.

Additionally, we found that patient age and smoking status were significant outcome predictors. Both predictors have previously been described in case of NPC.^37^, ^38^, ^39^, ^40^ The negative impact of patient age might be attributed to more comorbidities and therefore to an increased risk of death from other causes than NPC.^39^ Smoking can foster the occurrence or deterioration of various diseases, promote tumor growth, and reduce the efficacy of RCT.^37^, ^38^ Unfortunately, since the cause of death is unknown for a relevant proportion of our patients (6 out of 11 patients), it is difficult to draw further conclusions from our study. However, as reported by previous studies, our results indicate that elderly patients and smokers require special attention, possibly owing to reduced treatment efficacy and increased risk of side effects.^41^, ^42^

In our study, 53% of patients experienced relevant (≥III°) acute toxicity. The acute toxicity rates can be compared to recent studies.^7^ In terms of patient quality of life, late toxicity is more relevant.^43^ In our study, 27% of patients experienced relevant (≥II°) late toxicity. In particular, xerostomia (23% of patients, III° in 2 patients) was an important side effect, in accordance with previous reports.^44^ There were no cases of other possible severe side effects (e.g., temporal lobe necrosis with neurologic symptoms), possibly due to the high percentage of patients (67%) with modern IMRT or VMAT irradiation techniques.^44^, ^45^ Additionally, the rates of incomplete treatment application (radiotherapy: *n* = 2 [6.7%]; concomitant chemotherapy: *n* = 2 [7.4%]) were absolutely acceptable. Overall, our results indicate a good tolerability and feasibility of the NPC‐GPOH trials regimen in adults, which is in line with previous results from the study group.^12^

The presented study has limitations which have to be mentioned. Firstly, in a retrospective study with a small number of patients, the treatment was performed during a long time period which implies different lengths of follow‐up, and different irradiation techniques. Second, patient selection for each component of multimodal treatment (e.g., induction chemotherapy and adjuvant IFN‐β application) was carried out nonrandomized by different physicians, based on individual patient characteristics according to respective treatment patterns. These factors represent biases for the study results. Consequently, firm conclusions concerning the influence of the induction chemotherapy or the IFN‐β application on outcomes cannot be drawn. Additionally, since only one patient received the radiotherapy dose of 54 Gy after complete response to induction chemotherapy, we cannot draw further conclusions on oncologic outcomes with reduced radiotherapy dose in NPC patients. Thus, we are not able to clarify whether these specific components of the pediatric protocol of the NPC‐GPOH trials are beneficial in adult patients. Lastly, the lack of patient‐reported quality of life (beyond physician‐reported side effects) is a shortcoming of our study.^43^, ^46^ However, since the vast majority of NPC cases occur in the endemic regions of Asia and Africa, reports on treatment results throughout Europe are very rare (e.g., in Germany: Roeder et al.^47^ and Saleh‐Ebrahimi et al.^48^). Furthermore, reports on the NPC‐GPOH treatment regimen results are very rare.^7^, ^12^, ^13^, ^14^ Thus, overall, our study's results add relevant new findings to a poorly studied subject of research.

## 5 CONCLUSIONS

In adult patients, we found excellent outcomes and good feasibility of the NPC‐GPOH trials regimen. Additionally, we found evidence for patient subgroups with a special need to improve outcomes (smokers, WHO type I tumors) and with particularly excellent outcomes (WHO type III tumors). This stimulates further studies on treatment intensification or de‐escalation aiming at reduced side effects with optimal tumor control in NPC. As a suggestion, future studies could reduce treatment intensity (e.g., radiotherapy dose reduction) in patients with WHO type III tumors. An intensified treatment (e.g., systemic treatment) seems reasonable in patients with WHO type I tumors. In the light of recent studies, the EBV viral load at diagnosis and/or after RCT could serve as a valuable tool for tailored treatment (e.g., patient selection for adjuvant IFN‐β application). Additionally, further recently introduced immunotherapeutics^49^ could improve treatment outcomes.

## AUTHOR CONTRIBUTIONS

**Martin Leu:** Conceptualization (equal); data curation (equal); formal analysis (equal); project administration (equal); writing – original draft (equal); writing – review and editing (equal). **Hanibal Bohnenberger:** Data curation (equal); writing – review and editing (equal). **Manuel Guhlich:** Conceptualization (equal); project administration (equal); writing – review and editing (equal). **Markus Anton Schirmer:** Formal analysis (equal); writing – review and editing (equal). **Yiannis Pilavakis:** Data curation (equal); writing – review and editing (equal). **Hendrik Andreas Wolff:** Conceptualization (equal); data curation (equal); writing – review and editing (equal). **Stefan Rieken:** Formal analysis (equal); writing – review and editing (equal). **Leif Hendrik Dröge:** Conceptualization (equal); data curation (equal); formal analysis (equal); project administration (equal); writing – original draft (equal); writing – review and editing (equal).

## CONFLICT OF INTEREST STATEMENT

The authors declare no conflict of interest.

## ETHICS STATEMENT

The study was conducted according to the guidelines of the Declaration of Helsinki, and approved by the Ethics Committee of the University of Göttingen Medical Center (protocol code 9/6/19, June 20th, 2019).

## INFORMED CONSENT STATEMENT

This study was approved and the requirement to obtain additional informed consent was waived by the ethic committee of the University Medical Center of Göttingen (application number 9/6/19).

## Supporting information


**Figure S1.** Consort Diagram.

**Figure S2.** Disease‐free survival for the whole patient cohort.

**Figure S3.** Cancer‐specific survival for the whole patient cohort.

**Figure S4.** Locoregional control for the whole patient cohort.

**Table S1.** EBV status, IFN‐β application and histologic type.

## Data Availability

The datasets generated and/ or analyzed in the current study are available from the corresponding author by reasonable request.

## References

[1] Chua MLK , Wee JTS , Hui EP , Chan ATC . Nasopharyngeal carcinoma. Lancet. 2016;387:1012‐1024. 26321262 10.1016/S0140-6736(15)00055-0

[2] Downing NL , Wolden S , Wong P , Petrik DW , Hara W , Le QT . Comparison of treatment results between adult and juvenile nasopharyngeal carcinoma. International Journal of Radiation Oncology, Biology, Physics. 2009;75:1064‐1070. 19327901 10.1016/j.ijrobp.2008.12.030

[3] Kontny U , Franzen S , Behrends U , et al. Diagnosis and treatment of nasopharyngeal carcinoma in children and adolescents‐recommendations of the GPOH‐NPC Study Group. Klinische Pädiatrie. 2016;228:105‐112. 27135270 10.1055/s-0041-111180

[4] Barnes L , Eveson JW , Reichart P , Sidransky D . pathology and genetics of head and neck tumours, WHO classification of tumours. 3rd ed. IARC; 2005:84.

[5] Peterson BR , Nelson BL . Nonkeratinizing undifferentiated nasopharyngeal carcinoma. Head and Neck Pathology. 2013;7:73‐75. 23015393 10.1007/s12105-012-0401-4 PMC3597164

[6] Thompson LD . Update on nasopharyngeal carcinoma. Head and Neck Pathology. 2007;1:81‐86. 20614287 10.1007/s12105-007-0012-7 PMC2807508

[7] Buehrlen M , Zwaan CM , Granzen B , et al. Multimodal treatment, including interferon beta, of nasopharyngeal carcinoma in children and young adults: preliminary results from the prospective, multicenter study NPC‐2003‐GPOH/DCOG. Cancer. 2012;118:4892‐4900. 22359313 10.1002/cncr.27395

[8] Grüner A , Grabenbauer GG , Rödel C , et al. Nasopharyngeal carcinoma: only irradiation or simultaneous radiochemotherapy? Strahlentherapie Und Onkologie. 1999;175:591‐596. 10633784 10.1007/s000660050045

[9] Christiansen H , Wolff HA . Primary curative radiochemotherapy in nasopharyngeal carcinomas. Cumulative total cisplatin dose of prognostic significance. Strahlentherapie Und Onkologie. 2013;189:269‐270. 23319253 10.1007/s00066-012-0285-y

[10] Becker‐Schiebe M , Christiansen H . Update zur kombinierten Radio‐, Radiochemo‐ und alleinigen Chemotherapie bei der multimodalen Therapie des Nasopharynxkarzinoms—eine MAC‐NPC‐Metaanalyse. Strahlentherapie Und Onkologie. 2015;191:991‐993. 26459464 10.1007/s00066-015-0904-5

[11] Becker‐Schiebe M , Christiansen H . The best multimodal treatment for locally advanced nasopharyngeal carcinoma: An update of the MAC‐NPC data. Strahlentherapie Und Onkologie. 2017;193:856‐858. 28812097 10.1007/s00066-017-1189-7

[12] Mertens R , Granzen B , Lassay L , et al. Treatment of nasopharyngeal carcinoma in children and adolescents: definitive results of a multicenter study (NPC‐91‐GPOH). Cancer. 2005;104:1083‐1089. 15999363 10.1002/cncr.21258

[13] Römer T , Franzen S , Kravets H , et al. Multimodal treatment of nasopharyngeal carcinoma in children, adolescents and young adults‐extended follow‐up of the NPC‐2003‐GPOH study cohort and patients of the interim cohort. Cancers. 2022;14(5):1261. 35267570 10.3390/cancers14051261 PMC8909003

[14] Wolff HA , Rödel RM , Gunawan B , et al. Nasopharyngeal carcinoma in adults: treatment results after long‐term follow‐up with special reference to adjuvant interferon‐beta in undifferentiated carcinomas. Journal of Cancer Research and Clinical Oncology. 2010;136:89‐97. 19618214 10.1007/s00432-009-0640-2 PMC2779341

[15] Shanmugaratnam K , Sobin LH . The World Health Organization histological classification of tumours of the upper respiratory tract and ear. A commentary on the second edition. Cancer. 1993;71:2689‐2697. 8453591 10.1002/1097-0142(19930415)71:8<2689::aid-cncr2820710843>3.0.co;2-h

[16] Wolff HA , Bosch J , Jung K , et al. High‐grade acute organ toxicity as positive prognostic factor in primary radio(chemo)therapy for locally advanced, inoperable head and neck cancer. Strahlentherapie Und Onkologie. 2010;186:262‐268. 20437012 10.1007/s00066-010-2136-z

[17] Al‐Sarraf M , LeBlanc M , Giri PG , et al. chemoradiotherapy versus radiotherapy in patients with advanced nasopharyngeal cancer: phase III randomized intergroup study 0099. Journal of Clinical Oncology. 1998;16:1310‐1317. 9552031 10.1200/JCO.1998.16.4.1310

[18] Trotti A , Colevas AD , Setser A , et al. CTCAE v3.0: Development of a comprehensive grading system for the adverse effects of cancer treatment. Seminars in Radiation Oncology. 2003;13:176‐181. 12903007 10.1016/S1053-4296(03)00031-6

[19] NCI . Common Terminology Criteria for Adverse Events (CTCAE). v5.0 2024.

[20] Rubin P , Constine LS , Fajardo LF , Phillips TL , Wasserman TH , RTOG Late Effects Working Group. Overview . Late effects of normal tissues (LENT) scoring system. International Journal of Radiation Oncology, Biology, Physics. 1995;31:1041‐1042. 7713774 10.1016/0360-3016(95)00057-6

[21] Birgisson H , Wallin U , Holmberg L , Glimelius B . Survival endpoints in colorectal cancer and the effect of second primary other cancer on disease free survival. BMC Cancer. 2011;11:438. 21989154 10.1186/1471-2407-11-438 PMC3209454

[22] Jen C‐W , Tsai Y‐C , Wu J‐S , et al. Prognostic classification for patients with nasopharyngeal carcinoma based on American Joint Committee on cancer staging system T and N Categories. Therapeutic Radiology and Oncology. 2020;4:2.

[23] Pastor M , Lopez Pousa A , Del Barco E , et al. SEOM clinical guideline in nasopharynx cancer (2017). Clinical & Translational Oncology. 2018;20:84‐88. 29098554 10.1007/s12094-017-1777-0 PMC5785612

[24] Ribassin‐Majed L , Marguet S , Lee AWM , et al. What Is the best treatment of locally advanced nasopharyngeal carcinoma? An individual patient data network meta‐analysis. Journal of Clinical Oncology. 2017;35:498‐505. 27918720 10.1200/JCO.2016.67.4119 PMC5791836

[25] Luttke M , Spath R , Marschner S , Walter F . Induction chemotherapy with gemcitabine and cisplatin followed by radiochemotherapy in locally advanced nasopharyngeal carcinoma. Strahlentherapie Und Onkologie. 2020;196(8):740‐742. 32524161 10.1007/s00066-020-01644-6

[26] Zhang Y , Chen L , Hu GQ , et al. Gemcitabine and cisplatin induction chemotherapy in nasopharyngeal carcinoma. The New England Journal of Medicine. 2019;381:1124‐1135. 31150573 10.1056/NEJMoa1905287

[27] Yang SS , Guo JG , Liu JN , et al. Effect of induction chemotherapy in nasopharyngeal carcinoma: an updated meta‐analysis. Frontiers in Oncology. 2020;10:591205. 33489889 10.3389/fonc.2020.591205 PMC7820771

[28] Stoker SD , Novalic Z , Wildeman MA , et al. Epstein–Barr virus‐targeted therapy in nasopharyngeal carcinoma. Journal of Cancer Research and Clinical Oncology. 2015;141:1845‐1857. 25920375 10.1007/s00432-015-1969-3 PMC11823716

[29] Ma BBY , Lim WT , Goh BC , et al. Antitumor activity of nivolumab in recurrent and metastatic nasopharyngeal carcinoma: An international, multicenter study of the mayo clinic phase 2 consortium (NCI‐9742). Journal of Clinical Oncology. 2018;36:1412‐1418. 29584545 10.1200/JCO.2017.77.0388 PMC5941615

[30] Chan AT , Teo ML , Lee WY , Kwan WH , Choi PH , Johnson PJ . The significance of keratinizing squamous cell histology in chinese patients with nasopharyngeal carcinoma. Clinical Oncology (Royal College of Radiologists). 1998;10:161‐164. 10.1016/s0936-6555(98)80057-5 9704177

[31] Wang HY , Chang YL , To KF , et al. A new prognostic histopathologic classification of nasopharyngeal carcinoma. Chinese Journal of Cancer. 2016;35:41. 27146632 10.1186/s40880-016-0103-5 PMC4857443

[32] Gao W , Li ZH , Chen S , et al. Epstein–Barr virus encoded microRNA BART7 regulates radiation sensitivity of nasopharyngeal carcinoma. Oncotarget. 2017;8:20297‐20308. 28423621 10.18632/oncotarget.15526 PMC5386763

[33] Caponigro F , Longo F , Ionna F , Perri F . Treatment approaches to nasopharyngeal carcinoma: a review. Anti‐Cancer Drugs. 2010;21:471‐477. 20124988 10.1097/CAD.0b013e328337160e

[34] Semrau S . In HPV‐associated oropharyngeal cancer, chemoradiotherapy with cisplatin is superior to bioradiotherapy with cetuximab in terms of overall survival. Strahlentherapie Und Onkologie. 2019;195:447‐449. 30789992 10.1007/s00066-019-01441-w

[35] Lee VH , Kwong DL , Leung TW , et al. The addition of pretreatment plasma Epstein–Barr Virus DNA into the eighth edition of nasopharyngeal cancer TNM stage classification. International Journal of Cancer. 2019;144:1713‐1722. 30192385 10.1002/ijc.31856

[36] Hui EP , Li WF , Ma BB , et al. Integrating Postradiotherapy plasma Epstein–Barr virus DNA and TNM stage for risk stratification of nasopharyngeal carcinoma to adjuvant therapy. Annals of Oncology. 2020;31:769‐779. 32217076 10.1016/j.annonc.2020.03.289

[37] Guo SS , Huang PY , Chen QY , et al. The Impact of smoking on the clinical outcome of locoregionally advanced nasopharyngeal carcinoma after chemoradiotherapy. Radiation Oncology. 2014;9:246. 25424191 10.1186/s13014-014-0246-y PMC4251838

[38] Ouyang PY , Su Z , Mao YP , et al. Prognostic impact of cigarette smoking on the survival of patients with established nasopharyngeal carcinoma. Cancer Epidemiology, Biomarkers & Prevention. 2013;22:2285‐2294. 10.1158/1055-9965.EPI-13-0546 24252872

[39] Zhang Y , Yi JL , Huang XD , et al. Inherently poor survival of elderly patients with nasopharyngeal carcinoma. Head & Neck. 2015;37:771‐776. 24115004 10.1002/hed.23497

[40] Liu H , Chen QY , Guo L , et al. Feasibility and efficacy of chemoradiotherapy for elderly patients with locoregionally advanced nasopharyngeal carcinoma: results from a matched cohort analysis. Radiation Oncology. 2013;8:70. 23521779 10.1186/1748-717X-8-70 PMC3643871

[41] Mo J , Hu X , Gu L , et al. Smokers or non‐smokers: who benefits more from immune checkpoint inhibitors in treatment of malignancies? An up‐to‐date meta‐analysis. World Journal of Surgical Oncology. 2020;18:15. 31959178 10.1186/s12957-020-1792-4 PMC6971889

[42] Szturz P , Vermorken JB . Treatment of elderly patients with squamous cell carcinoma of the head and neck. Frontiers in Oncology. 2016;6:199. 27630826 10.3389/fonc.2016.00199 PMC5006317

[43] McDowell LJ , Rock K , Xu W , et al. Long‐term late toxicity, quality of life, and emotional distress in patients with nasopharyngeal carcinoma treated with intensity modulated radiation therapy. International Journal of Radiation Oncology, Biology, Physics. 2018;102:340‐352. 30191868 10.1016/j.ijrobp.2018.05.060

[44] Yeh SA , Tang Y , Lui CC , Huang YJ , Huang EY . Treatment outcomes and late complications of 849 patients with nasopharyngeal carcinoma treated with radiotherapy alone. International Journal of Radiation Oncology, Biology, Physics. 2005;62:672‐679. 15936544 10.1016/j.ijrobp.2004.11.002

[45] Du T , Xiao J , Qiu Z , Wu K . The effectiveness of intensity‐modulated radiation therapy versus 2D‐RT for the treatment of nasopharyngeal carcinoma: a systematic review and meta‐analysis. PLoS One. 2019;14:e0219611. 31291379 10.1371/journal.pone.0219611 PMC6619803

[46] McDowell L , Corry J , Ringash J , Rischin D . Quality of life, toxicity and unmet needs in nasopharyngeal cancer survivors. Frontiers in Oncology. 2020;10:930. 32596155 10.3389/fonc.2020.00930 PMC7303258

[47] Roeder F , Zwicker F , Saleh‐Ebrahimi L , et al. Intensity modulated or fractionated stereotactic reirradiation in patients with recurrent nasopharyngeal cancer. Radiation Oncology. 2011;6:22. 21356126 10.1186/1748-717X-6-22 PMC3055828

[48] Saleh‐Ebrahimi L , Zwicker F , Muenter MW , et al. Intensity modulated radiotherapy (IMRT) combined with concurrent but not adjuvant chemotherapy in primary nasopharyngeal cancer—a retrospective single center analysis. Radiation Oncology. 2013;8:20. 23347410 10.1186/1748-717X-8-20 PMC3599603

[49] Jouin A , Helfre S , Bolle S , et al. Adapted strategy to tumor response in childhood nasopharyngeal carcinoma: the French experience. Strahlentherapie Und Onkologie. 2019;195:504‐516. 30963203 10.1007/s00066-019-01461-6

