# Supplementary figures and images for: Multimodal treatment according to the NPC‐GPOH trials in adult patients with nasopharyngeal cancer—Analysis based on a single‐center experience
Source: Cancer Rep (Hoboken). 2024 Aug 27;7(8):e2111. doi: 10.1002/cnr2.2111 (PMC11349452; doi:10.1002/cnr2.2111)

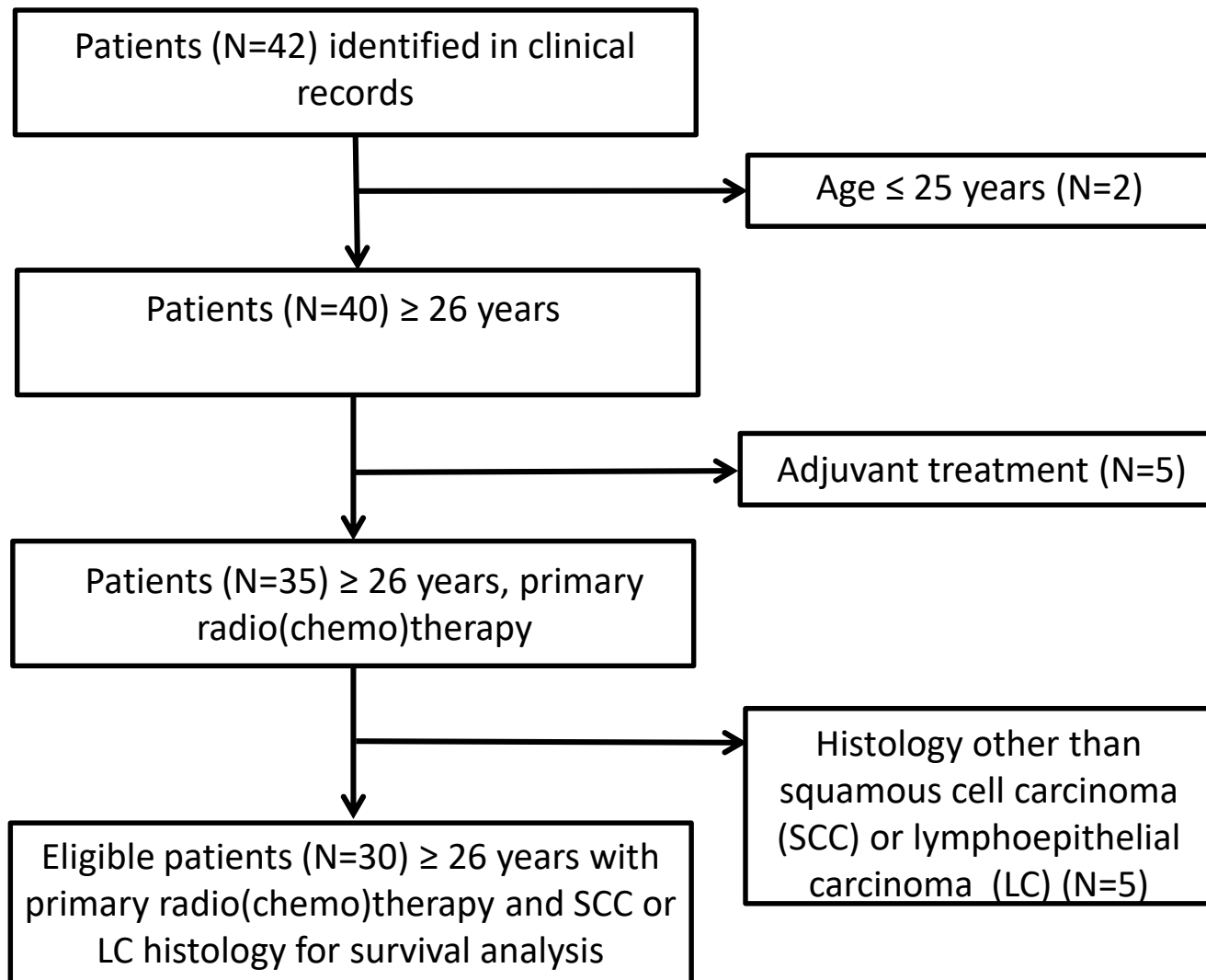

Figure S1. Consort diagram.

Supplement: Supplementary file 1 — Figure S1. Consort Diagram. [file CNR2-7-e2111-s002.pdf]

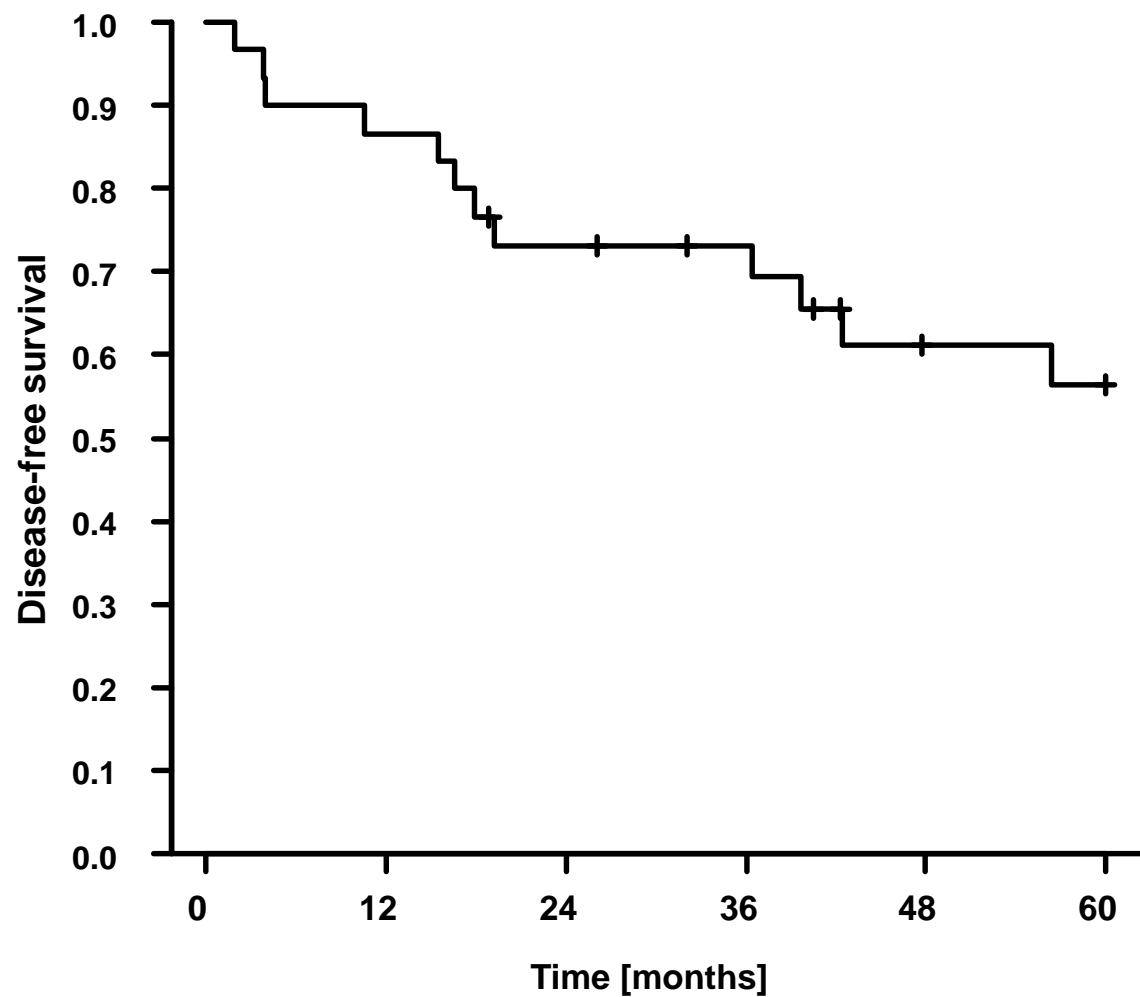

Patients at risk      26      21      19      13      12

Figure S2. Disease-free survival for the whole patient cohort.

Supplement: Supplementary file 2 — Figure S2. Disease‐free survival for the whole patient cohort. [file CNR2-7-e2111-s001.pdf]

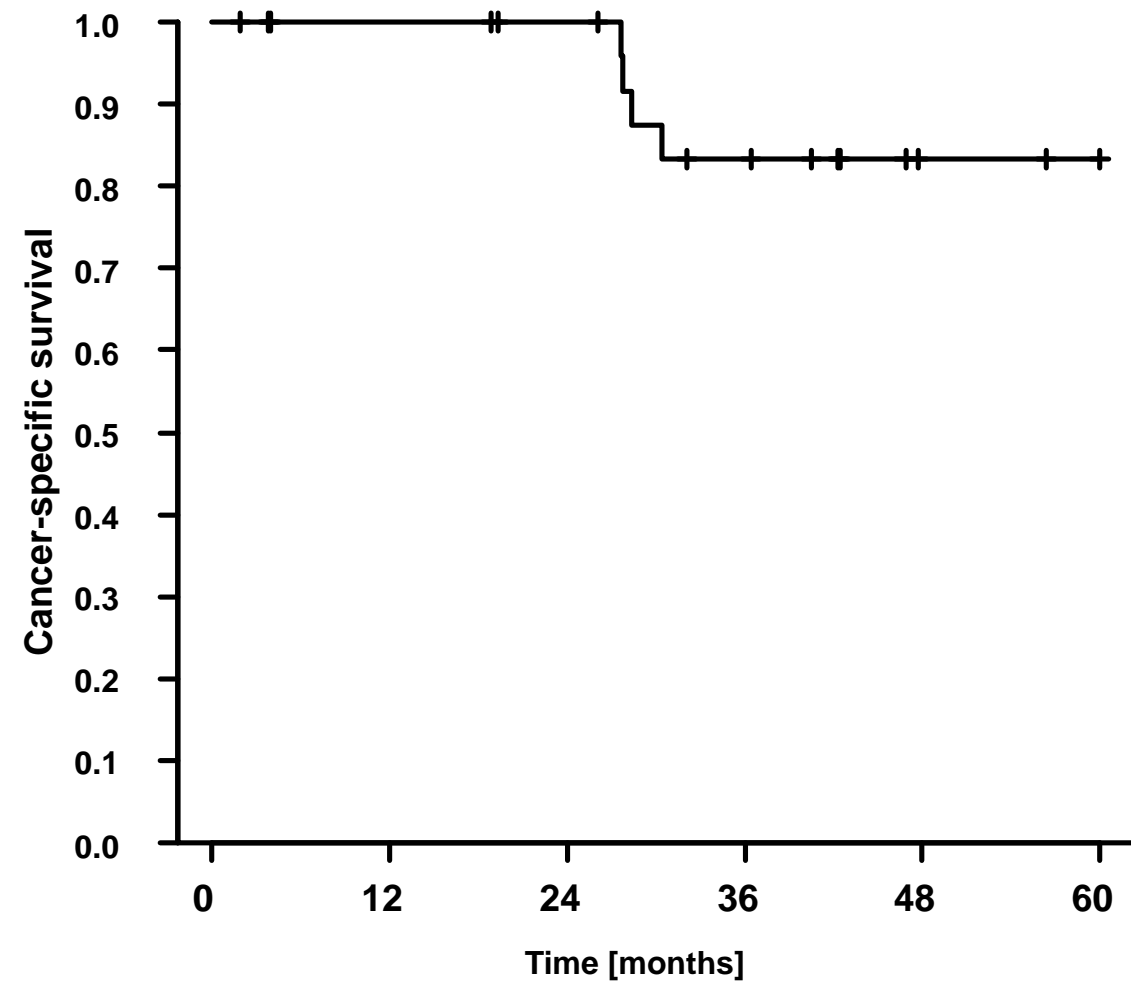

Patients at risk      27      25      19      13      12

Figure S3. Cancer-specific survival for the whole patient cohort.

Supplement: Supplementary file 3 — Figure S3. Cancer‐specific survival for the whole patient cohort. [file CNR2-7-e2111-s004.pdf]

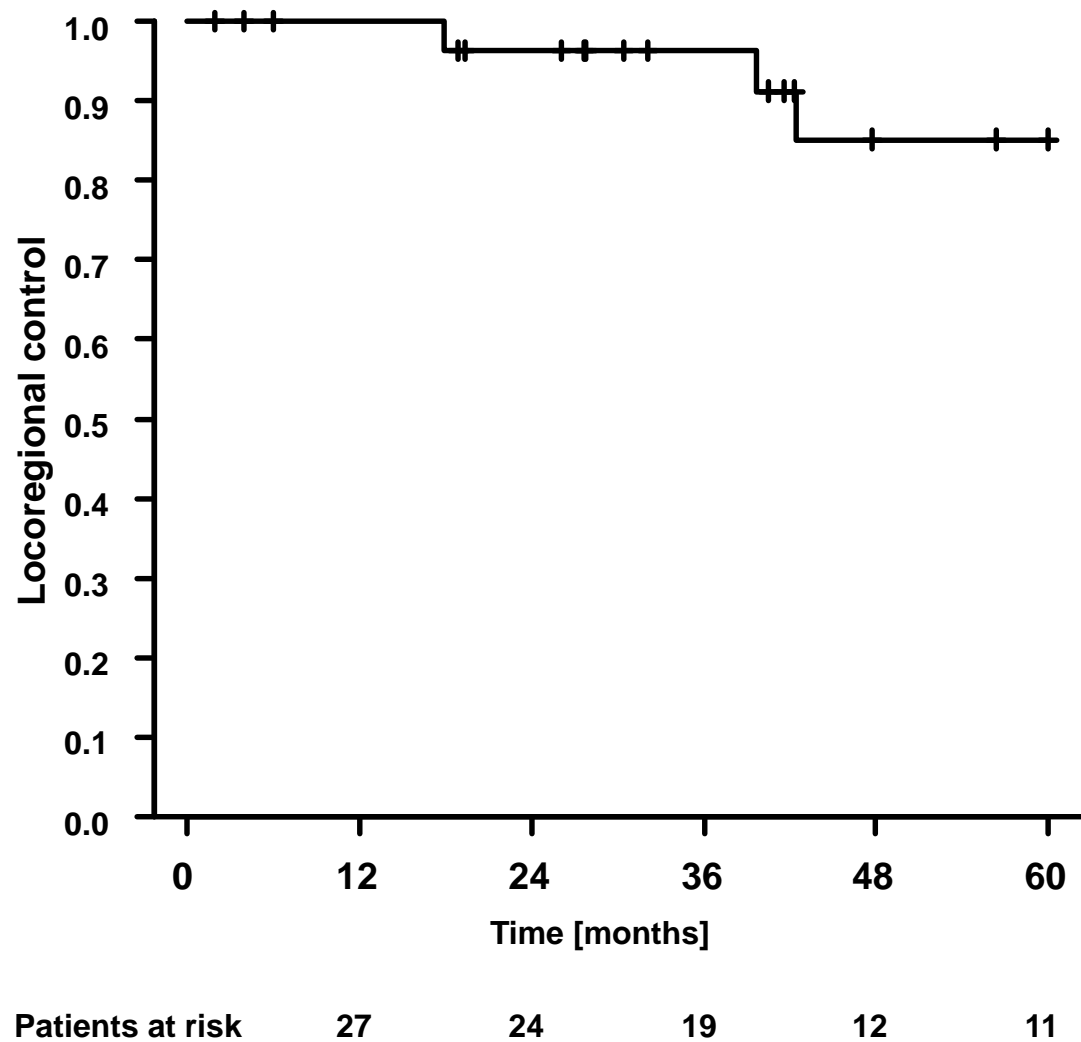

Figure S4. Locoregional control for the whole patient cohort.

Supplement: Supplementary file 4 — Figure S4. Locoregional control for the whole patient cohort. [file CNR2-7-e2111-s005.pdf]
