# Supplementary material for: Multimodal treatment according to the NPC‐GPOH trials in adult patients with nasopharyngeal cancer—Analysis based on a single‐center experience
Source: Cancer Rep (Hoboken). 2024 Aug 27;7(8):e2111. doi: 10.1002/cnr2.2111 (PMC11349452; doi:10.1002/cnr2.2111)
Supplement: Supplementary file 5 — Table S1. EBV status, IFN‐β application and histologic type. [file CNR2-7-e2111-s003.docx]

| **Table S1a.** EBV status, IFN-β application and histologic type | |
| --- | --- |
|  | All eligible patients (n=30), N (%) |
| EBV status |  |
| Positive |  |
| WHO type I | 2 (6.7) |
| WHO type II | 8 (26.7) |
| WHO type III | 11 (36.7) |
| Negative |  |
| WHO type I | 3 (10.0) |
| WHO type II | 5 (16.6) |
| WHO type III | 1 (3.3) |
| IFN-β application |  |
| yes |  |
| WHO type I | 0 (0.0) |
| WHO type II | 1 (3.3) |
| WHO type III | 8 (26.7) |
| no |  |
| WHO type I | 5 (16.7) |
| WHO type II | 12 (40.0) |
| WHO type III | 4 (13.3) |
| The number of patients and the percentage (in brackets) are given for each parameter. | |

| **Table S1b.** EBV status, IFN-β application and histologic type | | | | |
| --- | --- | --- | --- | --- |
|  | EBV positive (n=21) | EBV negative (n=9) | IFN-β application (n=9) | No IFN-β application (n=21) |
| WHO type I | 2 (9.5) | 3 (33.3) | 0 (0.0) | 5 (23.8) |
| WHO type II | 8 (38.1) | 5 (55.6) | 1 (11.1) | 12 (57.1) |
| WHO type III | 11 (52.4) | 1 (11.1) | 8 (88.9) | 4 (19.1) |
| The number of patients and the percentage (in brackets) are given for each parameter. | | | | |
